# Supplementary material for: Probing chromatin condensation dynamics in live cells using interferometric scattering correlation spectroscopy
Source: Commun Biol. 2024 Jun 24;7:763. doi: 10.1038/s42003-024-06457-2 (PMC11196589; doi:10.1038/s42003-024-06457-2)
Supplement: Supplementary file 2 — Supplementary Information [file 42003_2024_6457_MOESM2_ESM.pdf]

Supplementary Information

Probing Chromatin Condensation Dynamics in Live Cells Using Interferometric Scattering Correlation Spectroscopy

*Yi-Teng Hsiao[1], I-Hsin Liao[1,2], Bo-Kuan Wu[1], Hsueh-Ping Chu[2], and Chia-Lung Hsieh[1,3]**
[1]*Institute of Atomic and Molecular Sciences (IAMS), Academia Sinica, Taipei 10617, Taiwan*
[2]*Institute of Molecular and Cellular Biology, National Taiwan University, Taipei 10617, Taiwan*
[3]*Department of Physics, National Taiwan University, Taipei 10617, Taiwan*
*Email: clh@gate.sinica.edu.tw

Supplementary Note 1 Heterodyne DLS modeling for iSCORS measurement

The modeling of dynamic light scattering (DLS), also known as photon correlation spectroscopy, has been studied previously.[1, 2] The detection schemes can be categorized into homodyne detection and heterodyne detection. Homodyne detection measures the fluctuation of the scattering intensity of the particles without a reference beam, whereas heterodyne detection adds a coherent reference beam on top of the scattering intensity of the particles and thus measures the interferometric DLS signals. Consider a monodispersed nanoparticle sample, the general temporal autocorrelation function of the DLS signal ($G_2(\tau)$) can be written as[1, 2]

$$G_2(\tau) = \langle I(t)I(t+\tau)\rangle = \underbrace{(\overline{I_r} + \overline{I_s})^2}_{\text{background}} + \underbrace{2\overline{I_r}\,\overline{I_s} exp\left(-\frac{|\tau|}{\tau_C}\right)}_{\text{heterodyne term}} + \underbrace{\overline{I_s}^2 exp\left(-\frac{2|\tau|}{\tau_C}\right)}_{\text{homodyne term}} \qquad \text{Eq. (R1)}$$

Here, $I(t)$ denotes the time-varying intensity being measured, which is composed of the scattering signal of the particle, the reference beam, and their interference; that is $I(t) = I_s(t) + I_r(t) + 2\sqrt{|I_r||I_s|}cos\theta$, θ is the phase difference between the signal and the reference fields. $\overline{I_r}$ and $\overline{I_s}$ are the time-averaged intensities of the reference light and the scattered light from the sample, respectively, and τ_C is the correlation time. Based on the DLS theory, $\tau_C = \frac{1}{Dk^2}$ where D is the diffusion coefficient of the particles and k is the wavenumber of the measured scattering signal.

In iSCORS, the intensity of the reference beam is set to be much stronger than the scattering of the sample, i.e., $\overline{I_r} \gg \overline{I_s}$. Under this condition, the homodyne term becomes negligible. Thus, the normalized autocorrelation function of the DLS signal, denoted as $g_2(\tau)$, can be simplified as

$$g_2(\tau) = \frac{\langle I(t)I(t+\tau)\rangle}{\langle I(t)\rangle^2} = \underbrace{1}_{\text{background}} + \underbrace{C_s exp\left(-\frac{|\tau|}{\tau_C}\right)}_{\text{heterodyne term}} \qquad \text{Eq. (R2)}$$

where $C_s = 2\,\overline{I_s}/\overline{I_r}$.

In the image data analysis of iSCORS, the contribution of the static background, encompassing mainly the reference light intensity, is digitally subtracted through image postprocessing. Moreover, to estimate the diffusion coefficient D, we normalize Eq. (R2) with C_s and reduce it into a single exponential decay (as the form of Eq. (5) in the main text):

$$ACF_{DLS}(\tau) = \frac{g_2(\tau)-1}{C_s} = e^{-Dk^2\tau} \qquad \text{Eq. (R3)}$$

In conventional DLS, the signal detection is at the designated scattering angle, thus k is well-defined. In iSCORS, the effective k is determined by calibration experiments using monodispersed silica nanoparticle colloids with known sizes and diffusion coefficients (data shown in Supplementary Fig. 4). Given the k of our iSCORS microscopy, one can determine the D based on the decay rate of the $ACF_{DLS}(\tau)$. We note that the dot-shaped attenuator placed in the Fourier plane modulates the optical transfer function of iSCORS, and the effective k of iSCORS is affected by the shape of the attenuator.

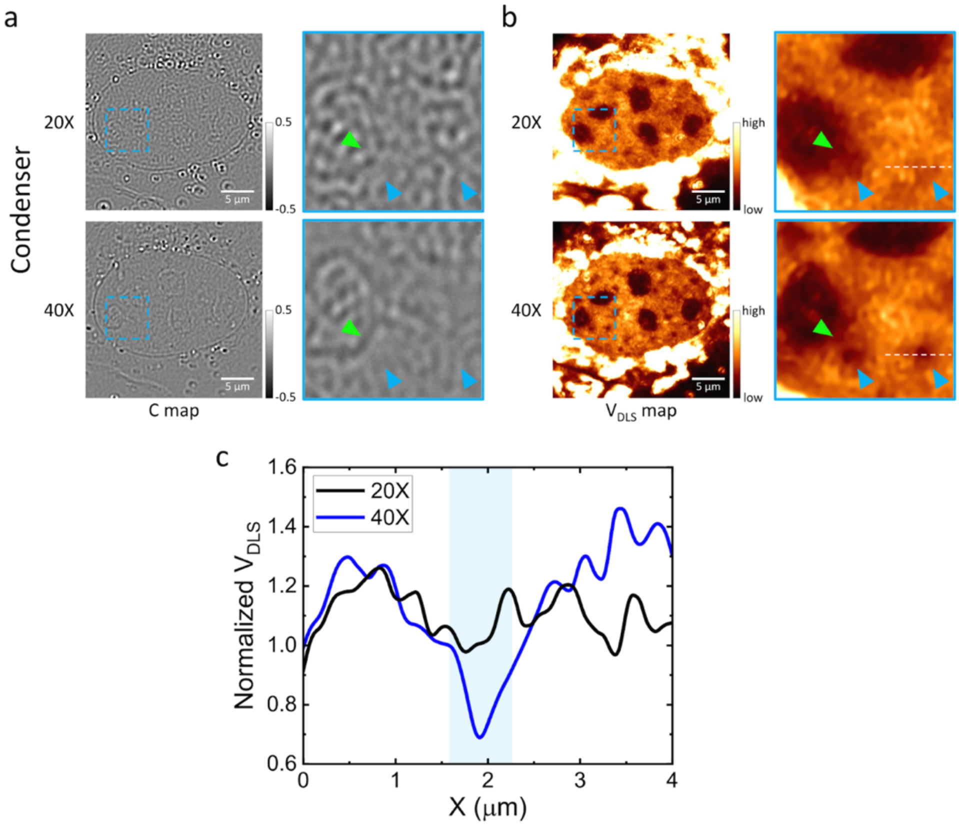

Supplementary Fig. 1 Enhancing the spatial resolution by reducing the spatial coherence of laser illumination

The spatial coherence of laser illumination was intentionally reduced by focusing the laser beam with a condenser lens and scanning it across the field of view. In this approach, the spatial coherence is determined by the size of the focused laser beam. Two condenser lenses, 20X and 40X water dipping objectives (UMPLFLN 20XW, NA0.5, Olympus and UMPLFLN 40XW, NA0.8, Olympus), were employed to assess the effect of condenser NA on spatial resolution. iSCORS images of a cell nucleus acquired using the two condensers are presented for direct comparison. (a) The nucleoli are more distinctly resolved using the 40X condenser in the C maps, compared to the 20X condenser. The green arrow indicates a clear boundary of nucleolus resolved with 40X condenser but barely resolved with 20X condenser. (b) In the V_{DLS} maps, the 40X condenser reveals small micron-sized chromatin-depleted regions with sharper boundaries and greater contrasts (indicated by the two blue arrows). (c) Intensity line profiles along the dashed lines in the V_{DLS} maps are displayed, illustrating enhanced contrast at the shaded area when utilizing the 40X condenser.

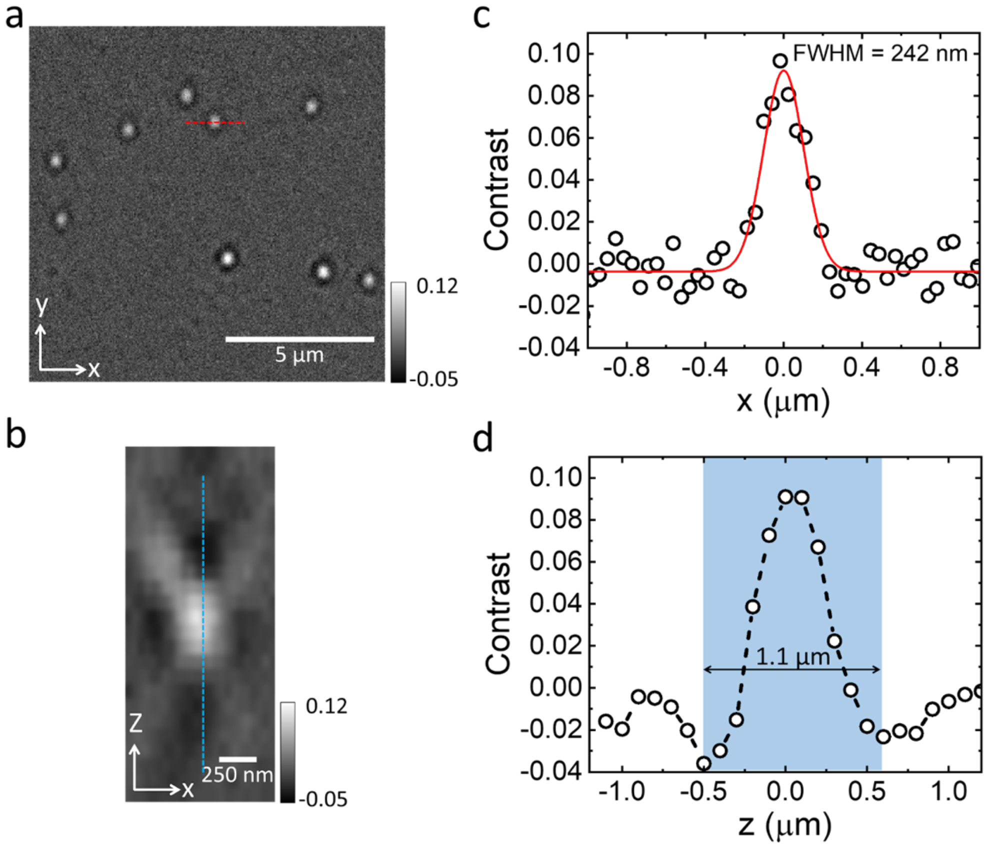

Supplementary Fig. 2 Point-spread function of iSCORS microscopy

We measure the point-spread function of iSCORS microscopy by imaging single 120-nm silica nanoparticles deposited on a clean coverglass (a). A z-stack image of a particle is acquired by moving the particle around the focal plane of the objective (b). The sectional views of the particle and their contrast line profiles in XY and XZ planes are displayed in (c) and (d), respectively. The red curve in (c) is a Gaussian fit, whereas the blue shaded area in (d) marks the axial range of the particle signal. The lateral and axial resolutions are estimated based on the widths of the blobs in XY and XZ, which are ~240 nm and ~1 µm, respectively.

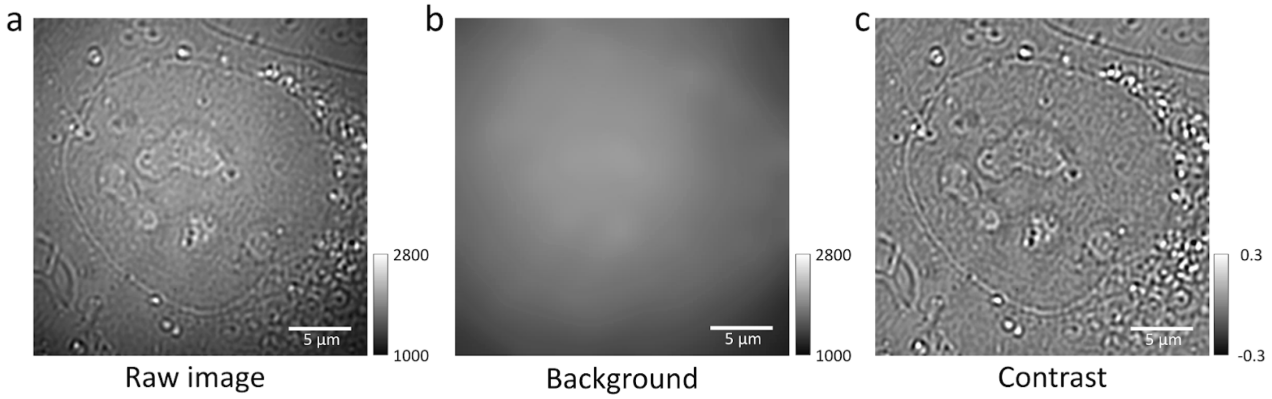

Supplementary Fig. 3 Flat field correction

We remove the slow varying illumination background through image post-processing. (a) A raw COBRI image of a cell nucleus. (b) Gaussian blurred image of the raw COBRI image with a standard deviation of 32 pixels (corresponding to 1.54 µm, approximately). (c) A background removed contrast map (C map) by normalizing the raw image with the gaussian blurred image.

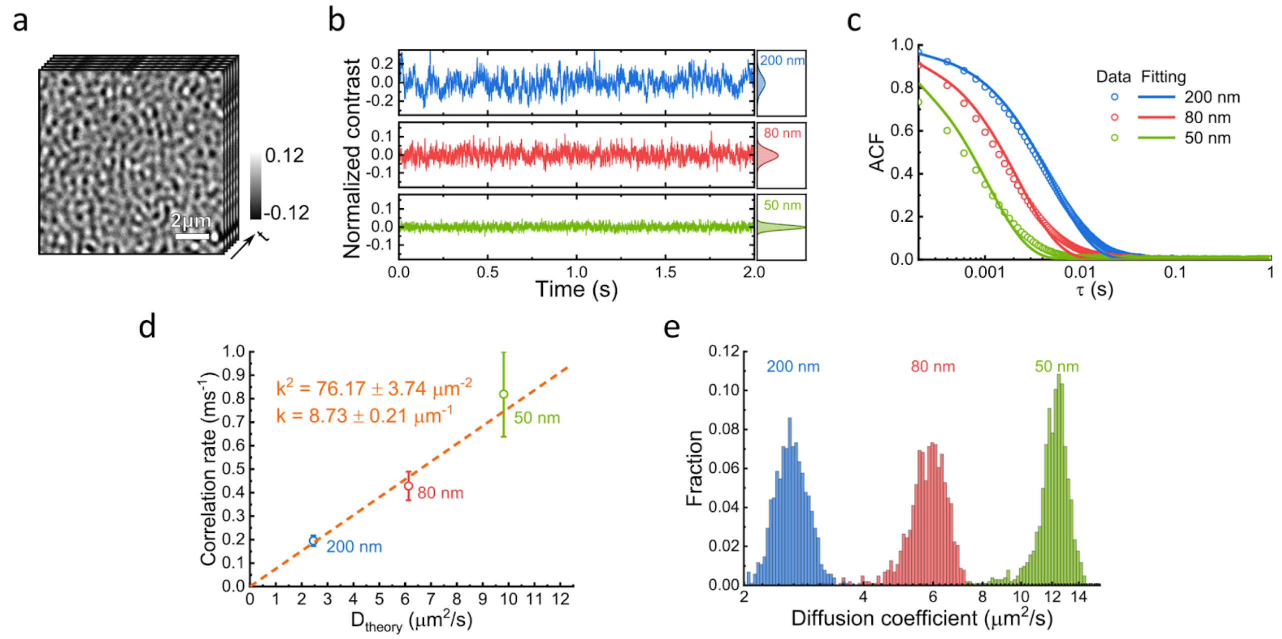

Supplementary Fig. 4 Quantitative measurement of *D* by iSCORS calibrated with nanoparticle colloids

We prepare monodispersed silica nanoparticle colloids with diameters of 50, 80, and 200 nm (NanoXact Silica Nanospheres, nanoComposix) for the calibration of diffusion measurement. The particle colloids were sealed in homemade chambers composed of two coverslips separated by double-sided tapes. At a particle concentration of 10 mg/mL, iSCORS microscope measures random speckle patterns of the sample due to the superposition of scattered fields of individual nanoparticles (as shown in (a)). Temporal varying interference contrast of each pixel and its temporal autocorrelation function ACF_{DLS} are calculated as described in the main text (depicted in (b) and (c), respectively).

We fit the $\mathrm{ACF}_{\mathrm{DLS}}$ with an exponential function:

$$\mathrm{ACF}_{DLS}(\tau) = e^{-\Gamma\tau}, \qquad \text{Eq. (R4)}$$

where Γ is the correlation rate. The measured ACF data agree well with the DLS model of exponential decay with a correlation rate Γ of 0.82 ± 0.18, 0.43 ± 0.06, 0.20 ± 0.02 ms^{-1} for the 50, 80, and 200 nm particle samples, respectively.

In the DLS model of freely diffusing particles,

$$\Gamma = Dk^2 \qquad \text{Eq. (R5)}$$

Where k is the effective optical wavenumber of the measurement.

In (d), we determine k as 8.73±0.21 $\mu\mathrm{m}^{-1}$ by correlating the measured correlation rates and the theoretical values of D based on Stokes−Einstein relation $D = k_B T / 6\pi\eta a$, where T=298K, η= 0.89 mPa.s of water, and a is the particle radius.

With the deduced k, we are able to measure quantitatively the D by analyzing the speckle temporal fluctuations of individual pixels. (e) plots the histograms of the measured D of the 50, 80, and 200 nm particle samples, which are 12.1±1.1, 5.8±0.65, 2.64±0.25 $\mu\mathrm{m}^2$/s respectively, agreeing well with the Stokes-Einstein relation of $D \propto a^{-1}$.

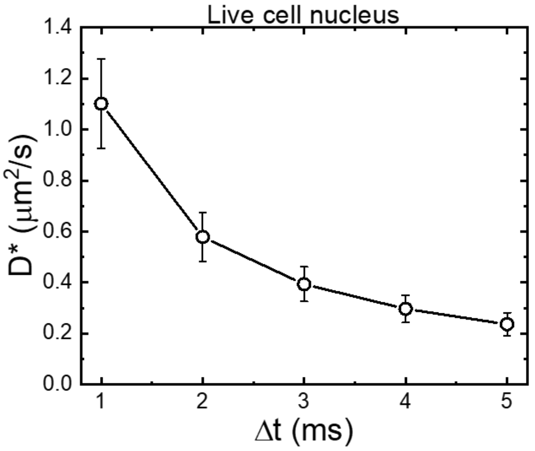

Supplementary Fig. 5 iSCORS measures a decreasing apparent diffusion coefficient (D^*) in live cell nuclei when the measurement timescale increases.

When performing iSCORS measureements in live cell nuclei at a timescale of 1 ms, a D^* of ~1 $\mu m^2/s$ is measured. Extending the iSCORS measurement timescale to 5 ms through downsampling the temporal data points causes D* to reduce by five-fold approximately. This indicates anomalous subdiffusion of chromatin, which leads to a slower apparent diffusion coefficient when measuring at a longer timescale.

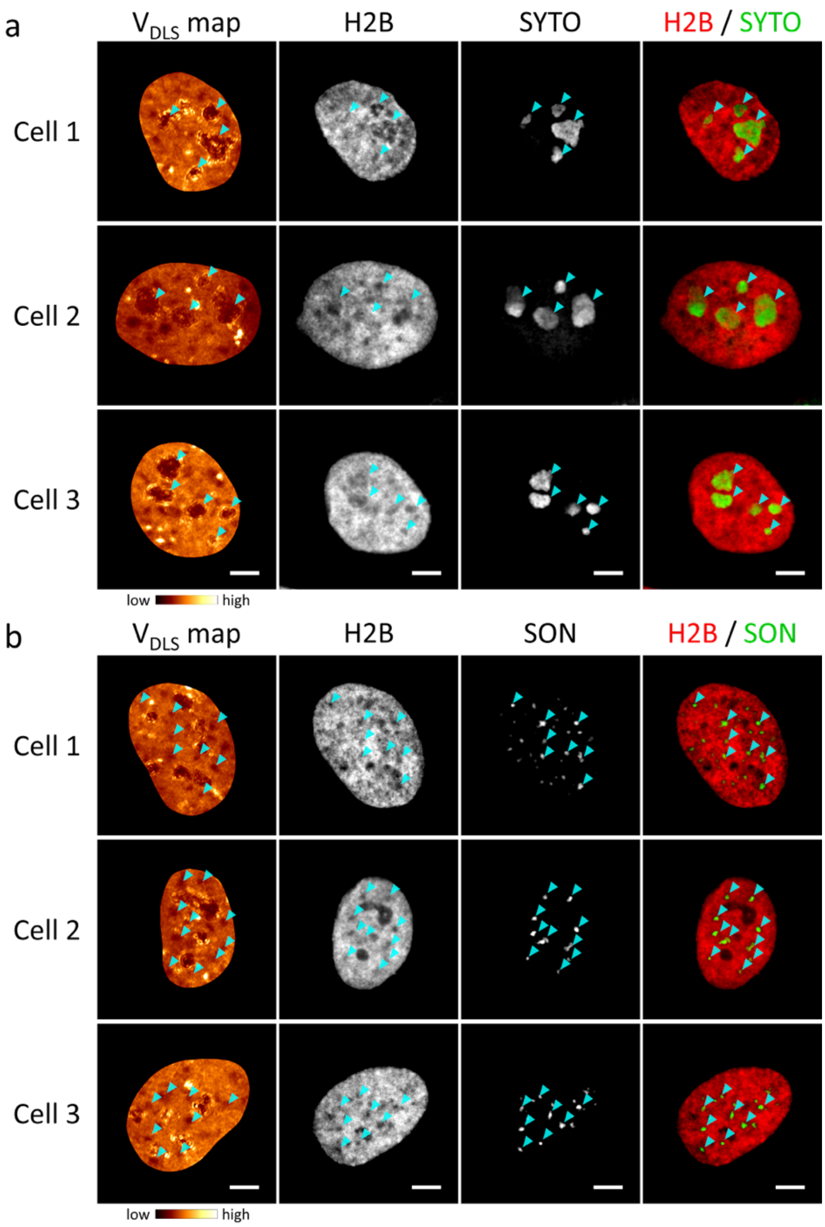

Supplementary Fig. 6 iSCORS signal is dominated by chromatin; nucleoli and nuclear speckles generate much weaker DLS signals.

Using fluorescence labeling against nucleoli and nuclear speckles, we examine the iSCORS signal intensity created by nucleoli and nuclear speckles, the major structures of RNA-protein complexes in cell nuclei. The live cell iSCORS imaging was performed on cells cultured on coverglass with grid markers. Then, the cells are chemically fixed and stained. Nucleoli are labeled by SYTO RNASelect, and nuclear speckles are marked by immunostaining against SON nuclear protein. The same cells are identified on a confocal microscope for fluorescence imaging. (a) V_{DLS} maps of representative cells, and corresponding confocal fluorescence images of chromatin (H2B-mCherry, red) and nucleoli (SYTO RNASelect, green). The locations of nucleoli are indicated by the blue arrows, exhibiting low chromatin fluorescence intensity and low V_{DLS} intensity. (b) V_{DLS} maps of representative cells, and corresponding confocal fluorescence images of chromatin (H2B-mCherry, red) and nuclear speckles (immunofluorescence against SON, green). The locations of nuclear speckles are indicated by the blue arrows, exhibiting low chromatin fluorescence intensity and low V_{DLS} intensity. Scale bars: 5 μm.

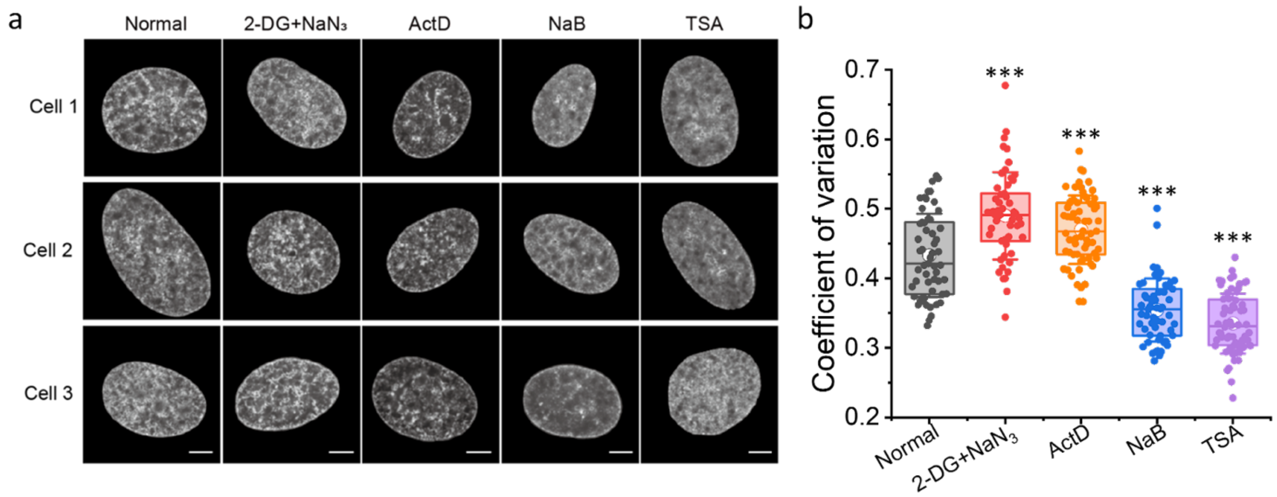

Supplementary Fig. 7 Effects of chemical treatments on global chromatin condensation levels determined through the analysis of Hoechst-stained fluorescence confocal images.

(a) Representative images of Hoechst-stained cells subjected to treatments that either promote chromatin condensation (2-DG+NaN_3 and ActD) or decondensation (NaB and TSA). The images reveal that fluorescence intensity displays greater spatial heterogeneity in cells with condensed chromatin. Conversely, the fluorescence appears more homogenous throughout the nuclei with decondensed chromatin. (b) The coefficient of variation (CV) for the Hoechst fluorescence across different treatments, quantifying the intensity fluctuations within a nucleus. For each nucleus, a CV value is defined as σ/μ, where σ and μ denote the standard deviation and the mean value of fluorescence intensity within the nucleus, respectively. Following this method, an enhanced CV value is measured for condensed chromatin, and a diminished CV value is measured for decondensed chromatin. Scale bars: 5 µm.

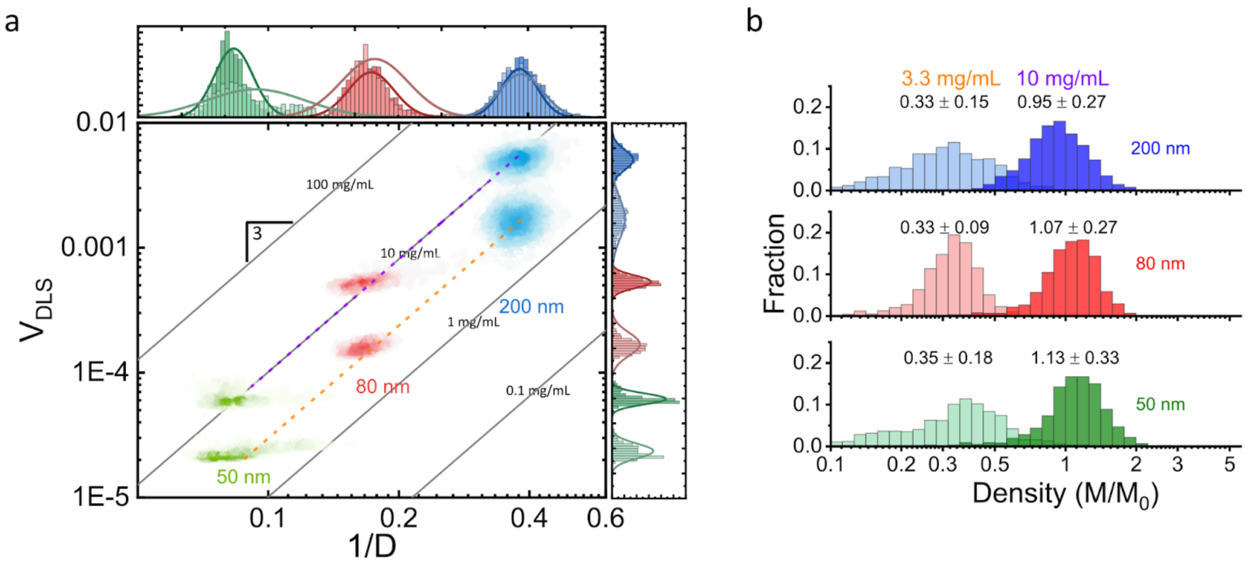

Supplementary Fig. 8 Characterization of mass density and particle size of nanoparticle colloids in a V_{DLS}-$1/D$ plot

Owing to the coherence nature of light scattering, the temporal variance of the time-varying interference contrast (denoted as V_{DLS}) is sensitive to the particle concentration and the particle size:[3]

$$V_{DLS} \propto Na^6, \quad \text{Eq. (R6)}$$

N is the particle number concentration, and a is the particle radius. Therefore, for a given particle size, V_{DLS} scales linearly with the particle mass density.

It is useful to examine the dependency of V_{DLS} on a when keeping the particle mass density constant because it mimics the scenario of chromatin condensation. With a constant mass density, $N \propto a^{-3}$, so $V_{DLS} \propto a^3 \propto (1/D)^3$. It manifests an interesting fact, that given the same particle mass density, a nanoparticle colloid of a larger particle size will generate a stronger V_{DLS}.

We validate this model by measuring the V_{DLS} and $1/D$ for 50, 80, and 200 nm particle colloids by iSCORS at two different concentrations, 3.3 mg/mL and 10 mg/mL, respectively (data shown in panel a). As expected, for a given mass density, the data of different particle samples fall on a line with a slope of three in the V_{DLS}-$1/D$ log-log plot, agreeing with the scaling of $V_{DLS} \propto (1/D)^3$. When changing the particle concentration, the data points move vertically in the V_{DLS}-$1/D$ plot, as V_{DLS} scales linearly with the mass density. Using this quantification based on V_{DLS} and $1/D$, the mass density of silica nanoparticle colloid can be accurately determined (panel b).

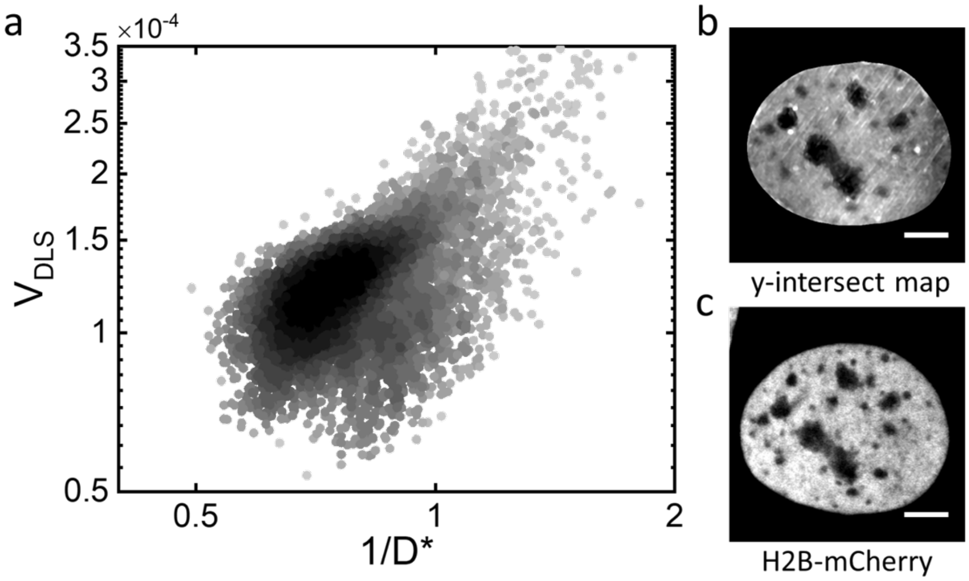

Supplementary Fig. 9 Correlation between the y-intercept map of a cell nucleus and the H2B-mCherry fluorescence image.

(a) A log-log plot of V_{DLS} and $1/D^*$ for each pixel within a cell nucleus. A line with a slope of three is constructed through each point to determine the y-intercept (not displayed), which is then assigned to the corresponding pixel. (b) The y-intercept map of the nucleus, displaying the y-intercept value of each pixel. It reveals distinct intranuclear structures, likely nucleoli and nuclear speckles. (c) Fluorescence confocal image of H2B-mCherry of the same cell nucleus, which closely aligns with the y-intercept map, confirming the correlation. Scale bars: 5 µm.

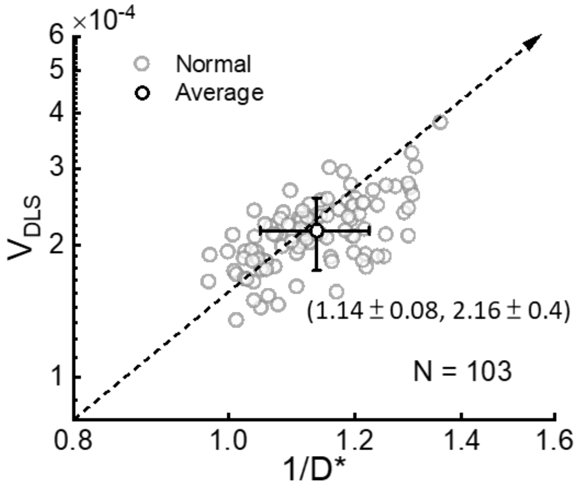

Supplementary Fig. 10 Distribution of chromatin mass density of individual U2OS cells estimated from the y-intersect values.

A spreading of V_{DLS} and $1/D^*$ is observed for individual cells, indicating the cell heterogeneity in chromatin configuration and our measurement errors. As illustrated with the data of more than 100 nuclei in a normal state, ~15% variations are observed between cells, which underscores our overall measurement uncertainty.

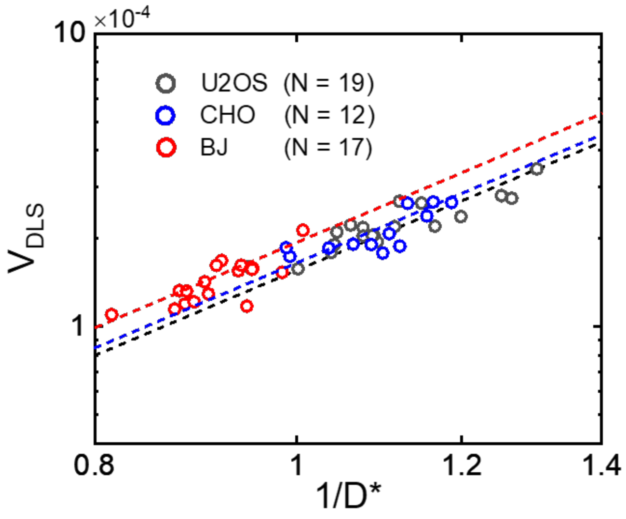

Supplementary Fig. 11 Chromatin mass densities of different mammalian cell lines are rather consistent.

iSCORS measurements were performed on three cell types: U2OS cells, Chinese Hamster Ovary (CHO) cells and Human foreskin BJ fibroblasts. The measured V_{DLS} and $1/D^*$ for individual cells are displayed on a log-log V_{DLS}-$1/D^*$ plot. A line with a slope of three is fitted to the measured data points for each cell type. The y-intersect values of the three lines, representing the mass densities of these three cell types, are rather consistent (variation of ~10%). This similarity suggests a conservation of chromatin density across mammalian cells, indicating potential similarities in the underlying mechanisms governing chromatin organization and dynamics. Further investigations are needed to explore potential differences in chromatin configurations in other cell types.

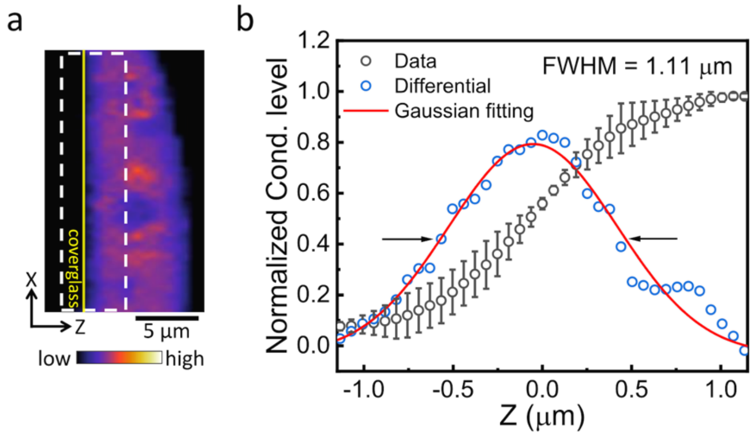

Supplementary Fig. 12 Axial resolution of chromatin condensation map.
The axial resolution of the chromatin condensation map is estimated based on the z-stack images of a cell cultured on a coverglass. The chromatin condensation level measured across the cell-coverglass interface serves as the line spread function. Its derivative with respect to z indicates the point spread function in z. (a) Cross-sectional view of a cell where the dashed square indicates the cell-coverglass interface. (b) Line profile for chromatin condensation level (black open circles) and its spatial differential (blue open circles). The red curve represents the Gaussian fitting to the point spread function, providing a FWHM of ~1.1 µm.

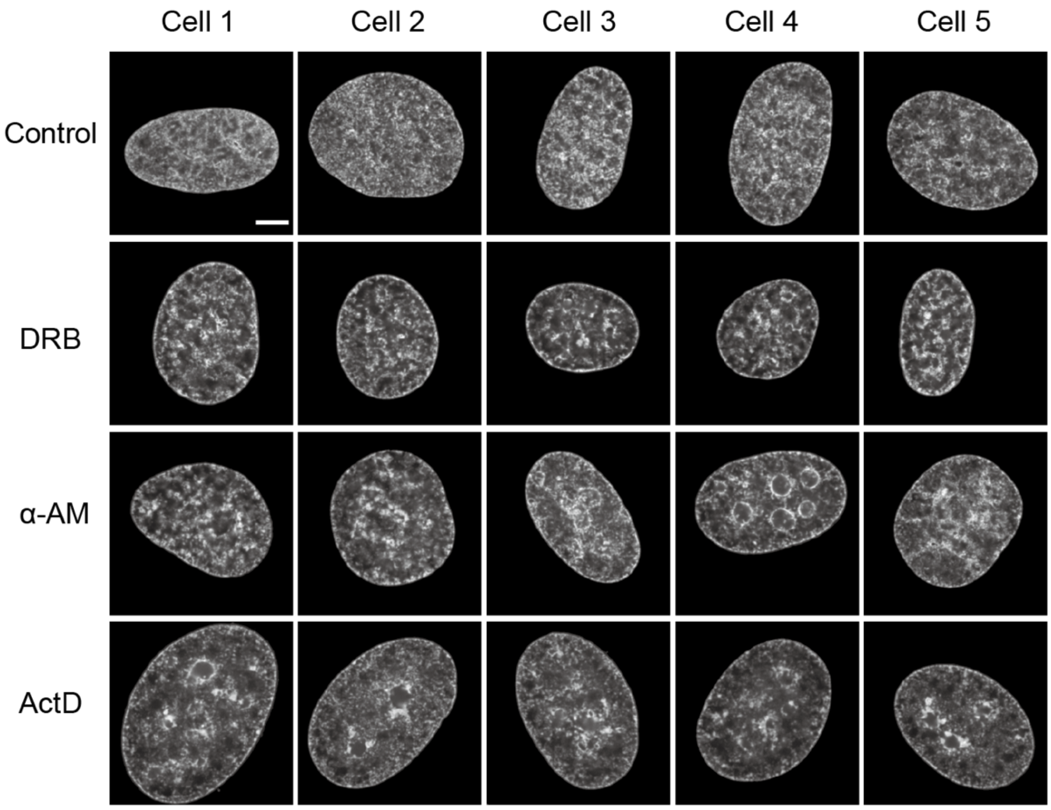

Supplementary Fig. 13 Additional image data of Hoechst fluorescence confocal images of cell nuclei under transcription inhibition.

Compared to the cell in the control group (top row), the Hoechst fluorescence intensities of the cell nuclei under treatments are more spatially heterogeneous, a signature of chromatin condensation. Such intensity variation can be quantitatively determined by calculating the deviation from the average intensity. Scale bar: 5 μm.

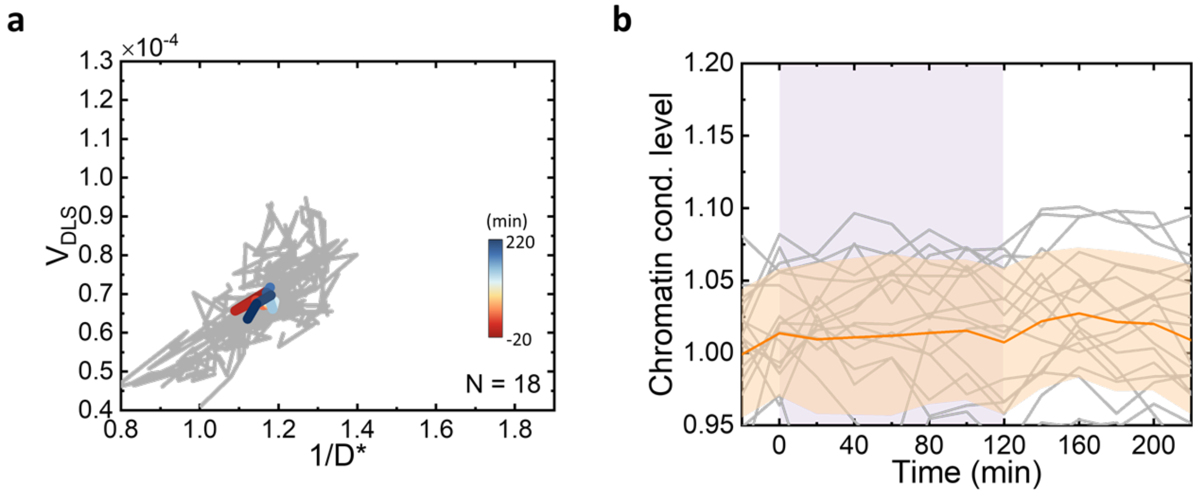

Supplementary Fig. 14 Control experiment of time-lapse iSCORS imaging with DMSO

(a) Trajectories of V_{DLS} and $1/D^*$ of 18 cells (gray) and their average (colored) displayed in a V_{DLS}-$1/D^*$ plot. DMSO is added at 0 minutes and removed at 120 minutes. (b) Chromatin condensation dynamics of the 18 cells (gray curves), and their average (orange curve) and standard deviation (orange shaded area). No noticeable changes in chromatin condensation level were observed with the addition of DMSO, supporting that the chromatin remodeling induced by DRB treatment is truly due to the inhibition of gene transcription.

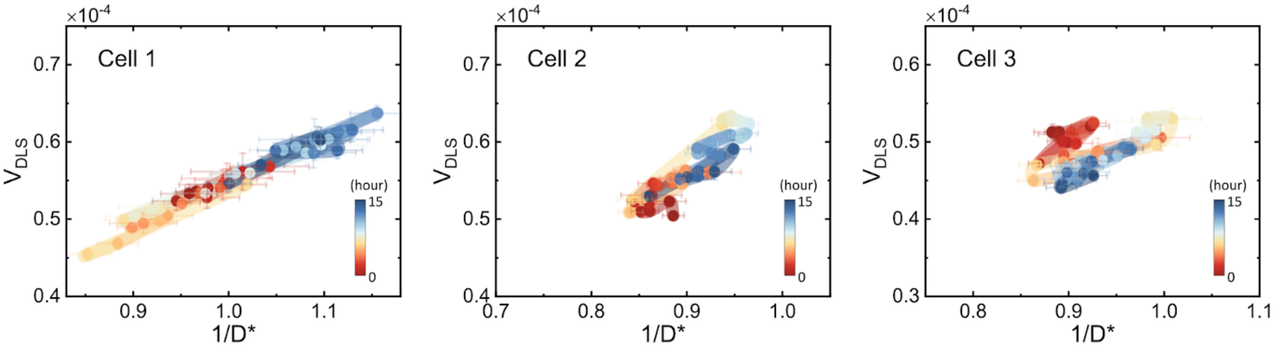

Supplementary Fig. 15 Long-term iSCORS data of single cell nuclei exhibiting different chromatin condensation dynamics.

Three live U2OS cell nuclei were continuously imaged by iSCORS for 15 hours, and their time-varying V_{DLS} and $1/D^*$ are displayed. While the data show positive correlations between V_{DLS} and $1/D^*$ that represent time-evolving chromatin condensation levels, it is evident that the three cells exhibit different chromatin condensation dynamics.

Supplementary References

1. de Jeu, W. H.; Madsen, A.; Sikharulidze, I.; Sprunt, S., Heterodyne and homodyne detection in fluctuating smectic membranes by photon correlation spectroscopy at X-ray and visible wavelengths. *Physica B: Condensed Matter* **2005,** *357* (1), 39-44.
2. Wu, W.; Knoll, M. S. G.; Giraudet, C.; Heinrich Rausch, M.; Fröba, A. P., Heterodyne dynamic light scattering for the characterization of particle dispersions. *Appl. Opt.* **2023,** *62* (30), 8007-8017.
3. Hsiao, Y.-T.; Tsai, C.-N.; Chen, T.-H.; Hsieh, C.-L., Label-free dynamic imaging of chromatin in live cell nuclei by high-speed scattering-based interference microscopy. *ACS Nano* **2022,** *16* (2), 2774-2788.

Supplementary Movie 1 Z stacks and cross-sectional views of iSCORS condensation maps and fluorescence confocal images of H2B-mCherry within a cell nucleus.

A series of iSCORS condensation maps is captured in a z-stack for a live U2OS cell nucleus by sequentially scanning the sample across the imaging plane. These maps display high axial resolution, revealing the three-dimensional organization of chromatin. The same cell is then imaged using a fluorescence confocal microscope, which captures the fluorescence from H2B-mCherry to delineate the 3D chromatin structures. Comparing the z-stacks from iSCORS and confocal fluorescence imaging reveals high spatial correlations, demonstrating not only the similar spatial resolutions offered by both imaging techniques but also the high specificity of iSCORS in detecting chromatin.
