# Supplementary material for: Probing chromatin condensation dynamics in live cells using interferometric scattering correlation spectroscopy
Source: Commun Biol. 2024 Jun 24;7:763. doi: 10.1038/s42003-024-06457-2 (PMC11196589; doi:10.1038/s42003-024-06457-2)
Supplement: Supplementary file 3 — Description of additional supplementary files [file 42003_2024_6457_MOESM3_ESM.pdf]

Description of Additional Supplementary Files

File name: Supplementary Data 1

Description: The source data behind the graphs in the paper.

File name: Supplementary Movie 1

Description: Z stacks and cross-sectional views of iSCORS condensation maps and fluorescence confocal images of H2BmCherry within a cell nucleus.
